# Assessment of effects of moon phases on hospital outpatient visits: An observational national study

**DOI:** 10.3934/publichealth.2023024

**Published:** 2023-05-06

**Authors:** Mohy Uddin, Aldilas Achmad Nursetyo, Usman Iqbal, Phung-Anh Nguyen, Wen-Shan Jian, Yu-Chuan Li, Shabbir Syed-Abdul

**Affiliations:** 1 Research Quality Management Section, King Abdullah International Medical Research Center, King Saud bin Abdulaziz University for Health Sciences, Ministry of National Guard - Health Affairs, Riyadh, Kingdom of Saudi Arabia; 2 Center for Health Policy Management, Universitas Gadjah Mada, Indonesia; 3 Health ICT, Department of Health, Tasmania, Australia; 4 Global Health and Health Security Department, College of Public Health, Taipei Medical University, Taipei, Taiwan; 5 International Center for Health Information Technology, Taipei Medical University, Taiwan; 6 Clinical Data Center, Office of Data Science, Taipei Medical University, Taipei, Taiwan; 7 Clinical Big Data Research Center, Taipei Medical University Hospital, Taipei, Taiwan; 8 Research Center of Health Care Industry Data Science, College of Management, Taipei Medical University, Taipei, Taiwan; 9 School of Hospital Health care Administration, Taipei Medical University, Taiwan. No 250 Wu-Hsing Street, Taipei 110, Taiwan; 10 Graduate Institute of Biomedical Informatics, College of Medical Science and Technology, Taipei Medical University, Taiwan. No 250 Wu-Hsing Street, Taipei 110, Taiwan; 11 Research Center of Cancer Translational Medicine, Taipei Medical University, Taiwan

**Keywords:** moon phase, lunar phase, hospital outpatient visit, human health, observational study

## Abstract

**Objectives:**

A vast amount of literature has been conducted for investigating the association of different lunar phases with human health; and it has mixed reviews for association and non-association of diseases with lunar phases. This study investigates the existence of any impact of moon phases on humans by exploring the difference in the rate of outpatient visits and type of diseases that prevail in either non-moon or moon phases.

**Methods:**

We retrieved dates of non-moon and moon phases for eight years (1st January 2001–31st December 2008) from the timeanddate.com website for Taiwan. The study cohort consisted of 1 million people from Taiwan's National Health Insurance Research Database (NHIRD) followed over eight years (1st January 2001–31st December 2008). We used the two-tailed, paired-t-test to compare the significance of difference among outpatient visits for 1229 moon phase days and 1074 non-moon phase days by using International Classification of Diseases, Ninth Revision, Clinical Modification (ICD-9-CM) codes from NHIRD records.

**Results:**

We found 58 diseases that showed statistical differences in number of outpatient visits in the non-moon and moon phases.

**Conclusions:**

The results of our study identified diseases that have significant variations during different lunar phases (non-moon and moon phases) for outpatient visits in the hospital. In order to fully understand the reality of the pervasive myth of lunar effects on human health, behaviors and diseases, more in-depth research investigations are required for providing comprehensive evidence covering all the factors, such as biological, psychological and environmental aspects.

## 1. Introduction

A vast amount of literature has been conducted in this field for investigating the association of different lunar phases with mental health, physical health, various diseases and human reproduction. The literature has mixed reviews in terms of association and non-association of diseases with lunar phases. Some studies have found the linkage of the birth month with neurological, reproductive, endocrine, immunological and inflammatory diseases in the lifespan [1]–[12]. Similarly, some studies have found a link between the moon and psychiatric, neurological and vascular illnesses [1],[2],[13]–[18]. On the other hand, some studies have found the non-association / non-correlation of lunar phases with the medical conditions ranging from cardiac arrest to mental pathologies [18]–[24]. Similarly, few detailed review and meta-analysis found insufficient or very less evidence to support the relationship between lunar cycles and human biology, birth, health aspects and other related activities [25]–[27]. Moreover, some studies assessing the influence of the moon on the seizure, ischemic stroke and heart attack have shown inconclusive results [18],[28]–[30].

With the advancement of technology, the existence of various online databases has enabled different data analysis, exploration and mining options. The availability of online databases, like dateandtime.com website [31], which deals with the time and dates related information, such as calendar, world clock, time zones, weather, sun and moon, and other calculators, has facilitated the time and dates based analysis. One of the important features provided by this database is the provision of data based on the lunar calendar. The scope of Electronic Medical Records (EMR) or Electronic Health Records (EHRs), which is the digital version of paper-based health information, varies globally [15]. Though the primary purpose of using EHR is to provide accurate, real-time and up-to-date patient information to the healthcare professionals in order to facilitate patient care and treatment; but secondarily EHR can also be used as an invaluable resource for supporting research, such as epidemiological, observational, safety and quality improvement research. In addition to the main feature of providing the administrative healthcare support, the Administrative Healthcare Databases (AHDB), such as Pharmacy and Health Insurance Databases, are also an important resource for health studies, e.g. population-based health monitoring and disease management studies. Though these systems are mainly used for billing and administration, they can also be used by researchers for mining the long-term data in order to see the impact of health interventions on healthcare systems in the real world [32]. Publically released in the year 2000, Taiwan's National Health Insurance Research Database (NHIRD) [33] is one of the largest administrative healthcare databases in the world and has been used for variety of research studies. By combining the above mentioned timeanddate.com database [31] and NHIRD [33], our research utilizes 1 million patients data covering time period of eight years (from 1^st^ January 2001 to 31^st^ December 2008) from NHIRD and dates of moon phases for eight years' time period (from 1^st^ January 2001 to 31^st^ December 2008). It investigates the existence of possible differences in the rate of outpatient visits and the type of diseases that prevail in lunar phases (“lluminated fraction < 50%” vs. “illuminated fraction >50%”). We refer to phases with illuminated fraction < 50% as ‘non-moon phases’ and phases with illuminated fraction > 50% as ‘moon phases’. To the best of our knowledge, this is the first of its kind study that provides observational evidence by following the rates of outpatient visits over lunar months with one of the largest sample size, i.e. 1 million cohort.

## 2. Methods

The methodology of this study is explained in the following three phases:

*Phase 1:* For this study, the third quarter and new moon phases were defined as ‘non-moon phase’, and the first quarter and full moon phases were defined as ‘moon phase’ [16]. We retrieved dates of moon phases for eight years of study period from January 2001–December 2008 from the website timeanddate.com. We excluded the lunar new year week (non-moon phase) and all the Sundays in the study period. The lunar new year starts on a new moon day (non-moon phase) with one-week vacation. The hospital outpatient clinics and general physicians' offices are closed for the entire week. This is followed by a slight rise in outpatient visits in the following week, which is the first-quarter period of the moon phase. Therefore, we have excluded one non-moon phase (Lunar new year week) and the following week (moon phase). However, Saturdays and bank holidays were included because outpatient clinics work half-day at that time, and there was also no significant difference between the number of bank holidays in the non-moon and moon phases. Table 1 shows the characteristics of the population's age and sex, and the number of visits per day in each phase. We used Chi-square test to compare observed number of hospital visits in the moon and non-moon phases. P-value indicates the statistical significance of the difference.

**Table 1.** Characteristics of the population and the number of outpatient visits.

	**Moon Phase**	**Non-Moon Phase**	**p-value**
**Sex**			
**Male**	487,787	486,614	0.592
**Female**	499,649	499,210	
**Age**			
**Mean**	33.03 (20.63)	33.07 (20.70)	<0.001
**Number of visits/day**	44,753	44,608	0.706

*Phase 2:* We analyzed the outpatient visits for 1229 moon phase days and 1074 non-moon phase days by using the International Classification of Diseases, Ninth Revision, Clinical Modification (ICD-9-CM) - 3 digit codes [31]. Our analysis included only the primary diseases (first disease code from the prescriptions) for each visit and diseases with over 1000 visits in the study period. As there were fewer days in the non-moon phase than in the moon phase, therefore we estimated the rate of outpatient visits per day for each primary disease. Since this is a secondary analysis of data, therefore no IRB approval was required.

*Phase 3:* We used the two-tailed, paired-t-test to compare the significance of the difference, if any, in the number of outpatient visits in both moon and non-moon phases for each disease, over 88 lunar months of the study period.

## 3. Results

**Figure 1.**
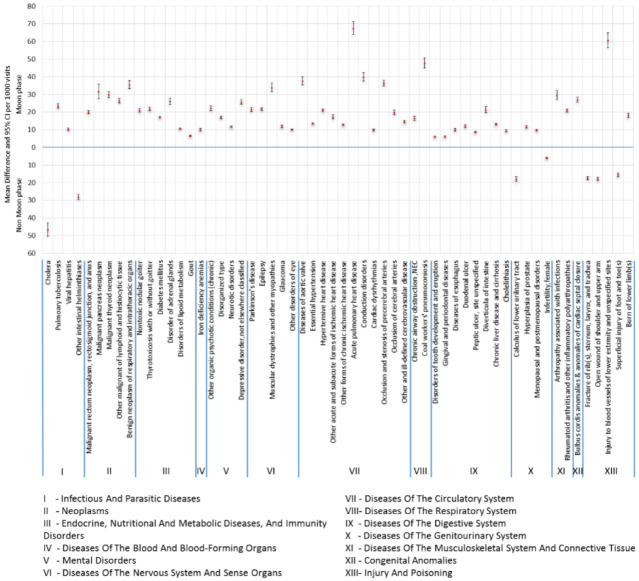
Showing mean difference and 95% confidence intervals per 1000 visits in non-moon and moon phases for 58 diseases with significant differences in the rate of outpatient visits over the 88 lunar months.

The results of the analysis showed that there were 58 diseases that showed statistical differences in number of outpatient visits in the non-moon and moon phases. We also estimated the mean difference per 1000 visits (see Figure 1). The results showed the diseases pertaining to ‘Acute Pulmonary Heart Disease’, ‘Injury to Blood Vessels of Lower Extremity and Unspecified Sites’ and ‘Coal Workers’ Pneumoconiosis' to be outstanding with a mean difference of 67.62, 60.65 and 47.76 respectively more visits in the moon phase. Meanwhile, ‘Cholera’, ‘Intestinal Helminthiases’ and ‘Disorders of Kidney and Ureter’ were outstanding in the non-moon phase with a mean difference of 46.60, 28.14 and 18.01 more visits, respectively.

## 4. Discussion

For the comparative analysis, the results from our study were compared with the previous related studies in this field. The results from the study conducted by Kazemi-Bajestani [34] using 5431 patient sample showed a slight increase for visits to psychiatric emergency room in the period of the full moon, as well as a significant increase in the severity of illness and aggressive behaviors in the start and end of moon cycles. In another study, Ahmad [2] investigated the link of lunar phases with medically unexplained stroke for the admissions of 7219 patients, found significant increase during the full moon phases, and reported no admission variation in the other significant days. But these individual studies do not appear to withstand scrutiny and meta-analyses show no effect. A different study from Finland [35] looked at the data involving 2111 male and 494 female victims from Oulu for suicidal occurrences during different lunar phases. The results revealed that there was a statistically significant gender difference during winter season in the lunar phases of suicides where full moon accounted for 40% of suicides in female and 24.3% in male. In addition, the association of full moon was statistically significant for premenopausal women that were younger than 45 years of age only. It raised the question that why only females (premenopausal females) showed the winter suicide peak at full moon, and acknowledged that universally there is no consensus for synchrony between the menstrual cycle and the moon phase.

Moreover, the study from Alhumoud [36] using 62,203 sample patients found that lunar cycle had no effects on hospital emergency room visits by mentally ill patients. Yang [37] using 559 patients analysis found that the environmental factors, such as full moon phase or supermoon event are not responsible for the increased visits to emergency departments for renal colic.

Although previous studies investigated the lunar phase effects on patients with specific conditions or diseases, e.g. Kazemi-Bajestani [34] and Alhumoud [36] looked at psychiatric patients, Yang [37] studied patients with renal colic disease, and Ahmad [2] considered stroke condition; they were having limited sample size. On the other hand, our study considered a wide domain of all outpatient visits during the time period of eight years, and the cohort consisted of 1 million population. Some aspects of a study from Boland [3] were similar to our work, as it had a large cohort, but it only evaluated the seasonality dependent disease risks effect based on the birth month, reported 55 significantly dependent diseases, and evaluated SNOMED-CT codes in terms of clinical contents. In comparison to that, our study reported 58 diseases, and showed statistical differences in non-moon and moon phases covering 88 lunar months in terms of clinical records, moreover our work was based on ICD-9 CM codes.

To the best of our knowledge, our study provided the first observational evidence by following the rates of outpatient visits over 88 lunar months with 1 million sample size. This study revealed the diseases which were prevalent either in the non-moon or moon phases. Figure 1 showed that diseases from all categories presented significant variations in outpatient visit rates in the moon phase. The utilization of 1 million cohort from NHIRD over the time period of eight years covers wide aspects of outpatient visits and diseases, therefore our study had certain limitations. The number of visits in the outpatient departments was extracted from NHIRD through e-Claims from Taiwan's hospitals / clinics. In general, the same patient could visit the hospitals multiple times in different phases of the moon, but we only considered the number of visits and reasons of the visits to the hospitals. For example, if a patient with hypertension visited the hospital 10 times a year, we counted the number of his visits in both moon phases and non-moon phases, and accumulated these differences for all the patients with hypertension visiting hospitals for eight years. We assumed that if there is no effect of moon phase, the number of visits will not be significantly different for eight years.

In our approach, we did not perform the Generalized Linear Mixed Models (GLMMs) and Generalized Estimating Equation (GEE) analysis methods because we were not claiming any casual relationships or effects of the moon, but were merely reporting the observations.

Though the patients' visits were held in multiple hospitals, but all hospitals had the same coding standards provided by NHIRD, as we used the ICD-9 CM codes. In addition, patients usually received 3 to 5 disease codes for each visit, where the first codes represented the reasons of the visits and other codes represented the co-morbidities or chronic conditions, e.g. if a diabetic patient visited the hospital with a lung problem, pulmonary heart disease would be the first code; therefore no code adjustment was required.

The moon's influence on the gravity, magnetic flux, atmospheric pressure, luminosity of the earth, and how moon affects human disease pathogenesis and behaviors, requires further in-depth investigation. These findings will reignite debate among researchers and clinicians as to find the truth about the myth of lunar effects on human health, behaviors and diseases.

We would also like to acknowledge that we excluded both the lunar new year holiday week and the following week. The lunar cycle is 29.53 days. There are 14.765 days of “non-moon phases” and 14.765 days of “moon phases”. By removing the new year week and the week following that, we removed 50% of the non-moon events during that cycle, but they removed only 47% of the moon events during that cycle. This procedure might introduce a substantial and systematic over-counting of “moon phase” events compared to “non-moon phase” events.

It is important to mention that due to our study limitations, we only considered the physiological changes / biological effect of lunar phases on the outpatient visits; but there are also other factors, such as psychological and environmental aspect that can have an impact on patients' health and behaviors along with diseases. The related literature also elucidated the influence of gravitational pull on the human body and other similar aspects in this field. Hence, the future studies should be conducted in order to understand all the triggering factors and to have a holistic picture. Similar to our future work suggestions, some review articles, e.g. Zimecki [40] also discussed that the exact mechanism through which the moon affects behavior and physiology is still not clear and has to be further investigated.

## 5. Conclusions

To the best of our knowledge, this unique study provided the first observational evidence by following the rates of outpatient visits over 88 lunar months using 1 million cohort and identified 58 diseases with significant variations through statistical differences. This data would allow other researchers to confirm that the counts are accurate, to independently compute the differences, and to perform independent statistical tests in the future. Finally, in order to fully understand this phenomenon, more in-depth research investigations are required that should provide comprehensive evidence by covering all the factors, such as biological, psychological and environmental aspects in this domain.
